# On human consciousness: A mathematical perspective

**DOI:** 10.1162/NETN_a_00030

**Published:** 2018-03-01

**Authors:** Peter Grindrod

**Affiliations:** Mathematical Institute, University of Oxford, United Kingdom

**Keywords:** Strongly connected delay networks, Bayesian nonbinary processing, Consciousness, Latent variables, Dual hierarchy

## Abstract

We consider the implications of the mathematical modeling and analysis of large modular neuron-to-neuron dynamical networks. We explain how the dynamical behavior of relatively small-scale strongly connected networks leads naturally to nonbinary information processing and thus to multiple hypothesis decision-making, even at the very lowest level of the brain’s architecture. In turn we build on these ideas to address some aspects of the hard problem of consciousness. These include how feelings might arise within an architecture with a foundational decision-making and classification layer of *unit processors*. We discuss how a proposed “dual hierarchy model,” made up from both externally perceived, physical elements of increasing complexity, and internally experienced, mental elements (which we argue are equivalent to feelings), may support aspects of a learning and evolving consciousness. We introduce the idea that a human brain ought to be able to reconjure subjective mental feelings at will, and thus these feelings cannot depend on internal chatter or internal instability-driven activity (patterns). An immediate consequence of this model, grounded in dynamical systems and nonbinary information processing, is that finite human brains must always be learning and forgetting and that any possible subjective internal feeling that might be fully idealized with a countable infinity of facets could never be learned completely a priori by zombies or automata. It may be experienced more and more fully by an evolving human brain (yet never in totality, not even in a lifetime). We argue that, within our model, the mental elements and thus internal modes (feelings) play a role akin to latent variables in processing and decision-making, and thus confer an evolutionary “fast-thinking” advantage.

## INTRODUCTION

Human consciousness presents a number of particularly baffling challenges to mathematics. On the one hand we know both the scale and the order of magnitude at which activity takes place in the human brain: there are billions of neurons, with each an active (excitable and refractory) electrical device, all coupled together within a characteristic network. These building blocks operate on the scale of cells, and their synaptic interactions. On the other hand it appears to be difficult to describe what kind of information processing the whole might achieve, and also how this furnishes human beings with the functionality usually associated with consciousness—including, at a very basic level, the ability of the organism to have feelings.

The theory of mind literature centers on defining aspects of what consciousness might be. It describes many such aspects, including the various properties of external perception, internal perception, intentionality, and the existence of free will. It says rather little about how these processes may actually occur, or their physical instantiation, or why these phenomena are so robust and reliable within the human species. How hard can it be, since they are so universally achieved?

Emergence, or emergent behavior, within coupled networks of (similar) interacting dynamical units (so called *complex systems*) refers to the appearance of macroscopic (higher domain) behavioral properties across such relatively large systems, often through robust phase change phenomena (as a function of scale or unit parameters, representing, say, operating conditions), that are often at first sight surprising or unfathomable from the perspective of a consideration of component elements in isolation (the lower domain). Goldstein (1999) defines emergence as “the arising of novel and coherent structures, patterns and properties during the process of self-organization in complex systems” (Introduction, para. 1). Philosophers generally term this *weak emergence*, since, though possibly *unexpected*, such properties may be demonstrated or deduced by very many, or some very complex, simulations and calculations, or analysis. Philosophers reserve the term *strong emergence* for properties that are beyond any such deduction (even in principle) based on knowledge of the low-level domain: In mathematical terms such phenomena must lie beyond any modeling and analysis. Most mathematical modelers would severely doubt if not reject outright that *any* such phenomena of real-world systems exist, but would rather assert that, in any particular instance, it is the present modeling paradigm (from concepts to models to equations to analysis) that fails to deduce the emergent properties, and thus it is not complete. Chalmers thinks the opposite, and asserts “there is exactly one clear case of a strongly emergent phenomenon, and that is the phenomenon of consciousness,” and says that “facts about consciousness are not deducible from any number of physical facts” (Chalmers, 2006, p. 3). In contrast, the aim of this paper is to examine the extent to which some properties of consciousness (at the high-level domain of *networks of networks* of neurons) are already deducible from modeling considerations, at the low-level domain of smallish strongly connected directed networks of interacting neurons.

There is however a rather widespread objection among philosophers to the genesis of consciousness as an emergent phenomenon because without further explanation it offers only a black box. For scientific modelers such a discussion of real information processing and consciousness is akin to watching a man try to eat a steak without a knife and fork: It must be swallowed whole or else left alone on the plate. This is not acceptable to them.

So to begin we shall introduce some suitable mathematical cutlery, including some relevant concepts from directed networks and dynamical systems that unlock behavioral functionality, and which allow us to make some predictions about the architecture that should drive effectiveness at low cost to the organism. We shall discuss some functional properties of relatively small systems that form the basic layers within the brain architecture. These have evolved specifically for real-time information processing and can underpin efficiencies in both perception (and hence reaction) as well as some aspects of consciousness. Instead of deploying “emergence” as the engine of consciousness, we propose to rely on an architecture of the processing units in making the successively sophisticated perceptions/interpretations (classification and decision-making) under incoming stimuli, and developing internal mental feelings in the process, that turn out to be advantageous.

We suggest these ideas impinge upon considerations as to how and why consciousness occurs (where do feelings come from?), and why this is useful (what is the role of feelings?).

In Chalmers (2010), the author makes a clear discrimination between the *easy problems of consciousness* and the *hard problem of consciousness*. In the early part in this paper we are nibbling away at some of the easy problems, perhaps in a way anticipated by Chalmers. These are explanations for attributes of consciousness that can, or ought to, be explained scientifically.

The hard problem is that of *experience*. What do we experience, what does it feel like, when we see and perceive things, actions, narratives? What is it like to be an organism that is perceiving the world? We will start out from a functional perspective by proposing an architecture that supports both physical and mental elements formed from the same underlying mechanisms (coupled dynamical ISGs) and arguing that the brain has a *dual hierarchy* of perception instantiated within a common network architecture. A dual hierarchy takes care of both the physical (external) elements that are perceived as well as the mental (internal) elements that are experienced. The latter will confer an evolutionary advantage by biasing immediate perceptions (reducing information requirements) and enabling fast and focused decision-making (at some cost to logic and rigour).

In the discussion of the hard problem of consciousness, the central point of this paper, we shall set out the conceptual structure (of the dual hierarchy model) in detail, assuming the functionality available from the earlier responses to the easy problem. We argue that the dual hierarchies (physical, external elements and mental, internal elements) are potentially infinite sets—there is always more to *know* and more to *experience*. And that each individual is on its own dynamical learning (and, sometimes, unlearning) curve. Hence consciousness is relative and evolving, depending on experience and exposure, and there is no universal level of achievement.

Our approach is essentially constructive. Emergence (weak) is confined to the smallish level of questions concerning what information processing activities can be achieved by small, strongly connected (irreducible) subgroups of neurons within the cortex. We build our understanding of the type(s) of global processing that may be carried out by a whole neuronal network lacing together many such small, strongly connected subgroups of neurons. The proposed dual hierarchy model, unlike black boxes, is thus specific and it may be explored, perturbed, and tested.

## PRELIMINARIES

### Architecture, Neurodynamics and Nonbinary Processing

**Figure 1.**
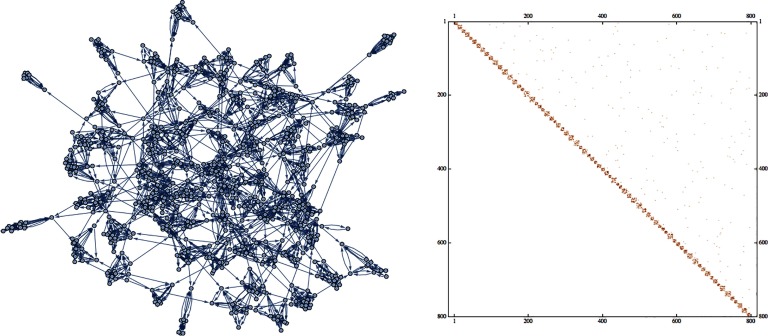
A directed network containing many ISGs, each distinct and of various sizes, together with its block upper triangular adjacency matrix (from Grindrod & Lee, 2017).

In a recent paper (Grindrod & Lee, 2017), the authors examined the behavior of *tight* bundles of coupled neurons, each having an excitable refractory dynamic. They assumed a global directed network consisting of a large sparsely connected array of much smaller, **irreducible subgraphs** (ISGs), representing directed neuron-to-neuron connections, each incurring some time delay that is relatively long when compared with the duration of a single neuron firing spike. Here “irreducible” simply means that there is a directed path between any ordered pair of neurons: Those subgraphs are sometimes called “strongly connected.” Irreducibility means that such subgraphs cannot be subdivided further into master and slave systems. Such an architecture is shown in Figure 1 (taken from Grindrod & Lee, 2017). Such a *modular* architecture was independently discussed in Meunier, Lambiotte, and Bullmor (2010).

In Grindrod and Lee (2017), it was assumed that the ISGs were *maximal* (that is, not part of any larger ISG) and therefore dynamically isolated from any downstream behavior: Thus their internal dynamics could be examined separately. It was also assumed that while the size *n* (the number of neurons) of such an ISG varied, the expected degree distributions of the in and out couplings remained constant, so that locally, on the inside around the neurons, all of the ISGs looked the same. Each neuron was assumed to have an excitable-refractory cell dynamic, and would hear any spike from any adjacent neuron, immediately upstream, after some (random) transmission time lag, longer than the timescale associated with an individual spike. There it was shown that the number of degrees of freedom exhibited by the (kick started and then) free running, autonomous dynamics of an ISG is distributed about a median (or mean) that grows only as fast as the logarithm of *n*. In free running each dynamical attractor (on the ISG’s *n* neurons) behaves generically like a winding map on a *k*-dimensional torus, and all this is happily independent of any particular choice of the ionic depolarizing dynamical system (such a Hodgkin-Huxley or FitzHugh-Nagumo; Grindrod, 1991); even a discrete excitable spiking and refractory process model will suffice (Grindrod & Lee, 2017). For each set of simulated ISGs, of a fixed size *n*, a value *k* may be estimated by either the use of embedding methods or directly from the Fourier spectrum.

A winding map on a *k*-dimensional torus is really just a *k*-dimensional clock (with *k* monotonically increasing phase variables). So *k* represents the number of degrees of freedom, and hence the number of different types of firing patterns, that the dynamical ISG may exhibit if it is periodically forced in the right ways.

The conclusion to be drawn from the modeling is that within an evolutionary efficient brain, which is constrained by both volume and energy, we should expect to observe architectures having very large numbers of smallish ISGs rather than occasional ISGs of arbitrarily large sizes or even giant components. And they must all be connected by a more loose macronetwork of inter-ISG connections. This situation is depicted in Figure 1.

Put simply, each ISG may be stimulated to behave in time in a number, *k*, of alternative modes, yet that number grows only sublinearly with its size. The modes themselves are dynamical patterns (across the ISG and over time) and they are competitive, one with another. They are not necessarily superposable, and when the ISG is stimulated periodically (from another upstream ISG) it may respond and become “resonant” with that particular input (in a suitably selected mode) or else display more disordered behavior. Thus the response of an ISG to upstream stimuli (and consequently the subset of its nodes might dominate) depends on where it is stimulated (which is the entry or receiving neuron) and the frequency of that stimulation. Only a single winning mode can emerge, damping or locking out the others, and when it does so only certain neurons may be heavily involved, resulting in the routing of stimulation to other ISGs that are immediately downstream of those participating neurons. Thus an ISG acts as an analog filter, a dynamical decision-maker (preferring one or another resonant mode), an amplifier, and a router. That is how ISGs process incoming stimuli to produce output information. ISGs are the building blocks for a layered information processor. By definition this processing is nonbinary, with multiple modes within each *processor* (each ISG) competing on the weight and repetitiveness of the stimuli, just as hypotheses might compete within a Bayesian multiple hypothesis updating process (Jaynes, 2003).

Bayesian multiple hypothesis testing essentially compares a set of alternate, mutually exclusive hypotheses against one another, with each new stimulus (an observation or piece of evidence) updating the odds on all hypotheses until one dominates. This enables decisions to emerge as evidence increases, starting out from a (subjective) prior expectation based on experience. This could enable, for example, the dynamic discrimination of the identity of various unknown sources (just as in the “Beatles or Stones” example given in Grindrod, 2015). The ISGs can perform a rough version of this without any calculations: The strength of each of the *k* alternative possible outputs (the patterns of firing) changes with new inputs (new upstream forcing), and they physically compete to dominate. This is a type of nonlinear resonance. Then the dominant pattern (dynamical state) of the ISG routes the information onwards, depending on which neurons are most involved and the frequencies of their firings.

To summarize, in Grindrod and Lee (2017) it is proposed that an evolutionarily efficient brain, maximizing the range of processing behavior available for given volume and energy constraints, would be in the form of a network or networks (many loosely networked smallish ISGs), and that the information processing is nonbinary, with each ISG able to exist within a number *k* >> 2 states. Moreover, the ISG can be reused to relay certain types of signals from distinct inputs through to corresponding outputs (for the right frequency of stimuli).

In Meunier et al. (2010) the authors advance a number of persuasive ideas supporting a hierarchical modular structure for information processing in the brain. These include arguments about locally segregated processing and efficiencies, timescale separations and stability, plausible mechanisms for development, and dynamic responsiveness to changing environments. To this list we can add the conclusions of the Grindrod and Lee (2017) experiments. Of course observable structure such as that in MRI data cannot capture the single neurons within ISGs, so that says little or nothing about the dynamical information processing taking place. Nevertheless, from both scales we can extrapolate to suggest the likely benefits of a hierarchical and modular structure.

Cascades and networks of winding dynamics over tori driving one another is a topic akin to that of coupled oscillatory systems where cascades of generalized clocks may drive one another in either type 0 or type 1 fashion (Glass & Mackey, 1988). Yet the generalization to tori and the classification of different types of entrainment is relatively unexplored (Grindrod & Patel, 2015).

Finally, we note that an information processor built on a nonbinary basic layer should be good at making decisions, yet probably poor (inefficient) in both logic and arithmetic. Binary computers are the complete reverse. Of course in principle one might build one from another (as in artificial *deep learning*), but not without requiring much capacity and much processing. The latter seems highly unreasonable and it should be horses for courses. Binary computers should do binary things, such as logic and arithmetic; nonbinary processors should do nonbinary things, such as multihypothesis decision-making based on partial information.

### Implications for Large-Scale Brain Simulation and Nonbinary Computing

The narrative and analysis given in Grindrod and Lee (2017) rests on the very large number of dynamical simulations of ISGs of sizes varying by orders of magnitude and stimulated in different ways. The assumption that there are (real valued) transmission delays that all are large compared with single pulse events is crucial in forcing the degrees of freedom exhibited to be large. If delays are merely integers, or else all delays are set to unity (as is sometimes the case in attempts to build arrays of linear or nonlinear nonbinary processors; Cassidy, Merolla, & Arthur, 2013), then the sophistication of the ISGs’ behavior simply collapses. That observation itself has clear implications for nonbinary computing platforms (such as McMillan, 2012) and for large-scale brain simulation programs (such as those discussed in Theil, 2015). The heterogeneity of the neuron-to-neuron connections is far more important than one’s particular choice of excitable-refractory neuronal dynamics. Ironically, the numerical solution of large systems of delay differential equations (with a plethora or real valued delays) is very expensive (in terms of both compute time and memory) to achieve on a standard binary computing device; yet it is easily achieved electrochemically when instantiated (hardwired by neurons) within the brain itself.

### Friston’s Bayesian Brain

The Bayesian brain is not a new idea, and indeed many of the constructs developed in Grindrod and Lee (2017) were foreshadowed by ideas and discussions set out by Friston (2013, and the references therein), coming from a completely different starting point. Instead of ISGs, Friston proposes active bundles of (implicitly strongly connected) neurons isolated within a Markov blanket. The dynamics of the active cells is expected to be complex (usually illustrated with chaotic, chattering attractors), whereas in Grindrod and Lee (2017), when left to free run, the ISGs result in winding dynamics over lowish dimension tori.

### Physical Structures

The cerebral cortex is the brain’s outer layer of neural tissue in humans and other mammals, which is wrapped over the limbic brain (which controls more basic functions). The cortex is usually asserted to play a role in memory, perception, thought, language, and consciousness.

The neocortex (the most recently developed part) is differentiated into a number of horizontal layers, and neurons within the different layers connect vertically to form small *circuits*, called cortical columns. It is these tightly connected columns that are highly suggestive of a massive number of ISGs, with each acting as a multimodal processor, yet integrated more loosely across the surface of the cortex. There is thus some physical evidence for the “network of networks” architecture that is suggested to be efficient here.

### Subconscious Heuristics

The rise of behavioral analytics and the demonstration of the use of a range of heuristics as a means of making fast, possibly biased, and certainly suboptimal subconscious decision-making (Ariely, 2008; Kahneman, 2011) are amply demonstrated by the human brain’s failure to reason (subconsciously) logically or quantitatively in even relatively simple circumstances. The fast-reasoning paradigm has been hugely successful in explaining a very wide range of human cognitive biases and illusions, including loss aversion, while hastening the brain’s response by massively reducing the cognitive effort required in any challenging circumstance. A by-product is the occurrence systematic biases (even cognitive illusions: “What you see is all there is;” Kahneman, 2011).

The evidence of apparent heuristics enabling fast, subconscious decision-making suggests that the most essential element that is required within any model of the brain’s information processing is that multiple hypotheses must compete based on both partial and missing information (the heuristics reflect this—they are observable consequences of it). The architecture and functionality discussed in Grindrod and Lee (2017) indicate that this can happen at the scale of a single ISG.

## WHAT WORK CAN THE MACHINERY OF THE CORTEX DO?

We have argued that each column (that is, each ISG) is a low-level decision-maker, where multiple hypotheses compete. When driven at some point of entry from upstream it either acts incoherently or locks into one of many modes. Consequently certain neurons within the column become relatively more involved and then information can propagate, through them, downstream on to other columns. Thus each column acts as a nonbinary processing filter and consequently a directional router for distinctive types of patterned behavior. What can a whole mesh of such objects achieve?

The most obvious response is that of searching for and distinguishing between different types of patterns. The human brain is remarkably good at this. And the same ability can be used for a range of distinct inputs. Thus brains should discriminate between visual patterns (just as we search naturally for patterns in random images or when we look at clouds) as well as audio patterns within both language and music. The latter are most interesting since they have been developed by humans to be learnable, pleasurable, and evocative. We may think of language as the *negative* image of the brain’s *positive* machinery. There are no languages that are difficult for human babies to learn; they have evolved structures that are intrinsically learnable and well suited to the brain’s machinery.

The machinery must be able to make a decision that certain patterns are indeed embedded (or not) within the data that match stored, remembered patterns, observed earlier and to which a label has been associated; or else the new data contain none of the previously known patterns, and thus could be discarded or used to create a new remembered template if there is a strong recurrent pattern within it. Thus the cortex needs to hold a lexicon of *motif* patterns as certain firing modes of certain ISGs, in time and across its columns, as well as being able to search out any one such pattern and to select that most likely to be dominant and hence associate the incoming stimuli with the mode. In doing so itself it might be best tactically to analyze the incoming data recursively, at multiple resolutions, both discarding what is inconclusive or commonplace and honing in on points of peculiarity/idiosyncrasy.

We suggest that the machinery might instantiate a (hardwired) methodology that tunes into a number of modes continuously that is similar, in a rough and ready way, to the expectation-maximization (EM) algorithm (Bishop, 2006; Grindrod, 2015), which provides a solution to finite mixture problems, where the totality of all incoming signals are described in terms of a finite mixture of signals from a number of distinct sources (that correspond to the distinct dynamical modes). It is important to note that though the brain may have evolved physically to instantiate and perform *like* the EM algorithm, because that is a very effective and efficient way of recognizing multiple sources within multiple signals, the brain itself contains no such embedded algorithm.

The EM algorithm and its generalizations may be quite unwieldily to program onto a standard digital computing platform; but we suggest that the physical machinery of the cortex (as a platform) may make this functionality relatively trivial, being only one level up from the basic functionality available at the ISG level.

## IMPLICATIONS FOR THE HARD PROBLEM OF CONSCIOUSNESS

What is it like to be an organism that is perceiving the world? Chalmers (2010) suggests that this problem is (and may remain) beyond deduction, beyond any scientific explanation. Here we shall introduce a conceptual model, building on the task-based processing and multimodal decision-making properties of the brain’s architecture. We will speculate that the successive sophistication of the *elements* that may be perceived is based on a *dual hierarchy*. Here we shall use the term *dual* to reflect the concept of dual spaces within mathematical analysis rather than mind-body dualism. Our purpose is to approach the hard problem by suggesting how feelings come about, using the processing units that we have discussed (the network of networks), and why they can play a useful role, conferring an evolutionary advantage.

We start with a simple hierarchy based on the successive integration and perceived understanding of external information (and upon stored contexts and expectations) dealing with physical elements, objects, events, and higher domain structures that are external to the brain. We shall set this up and then argue that internal elements (mental objects, events, feelings, and so on) must occupy a second hierarchy that is orthogonal to, or a dual of, the first hierarchy.

**Figure 2.**
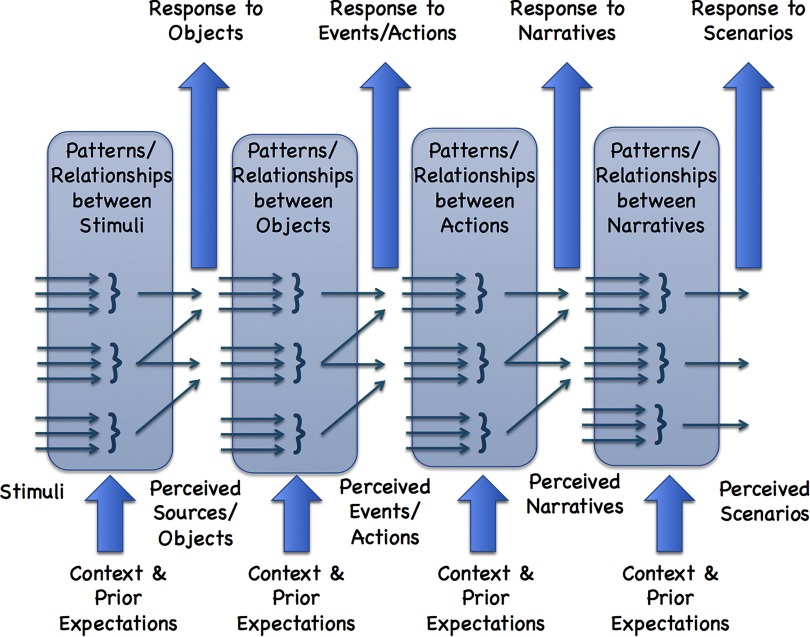
Hierarchical arrangements of recursively derived real-world (external) modal elements: From distinguished objects through to distinguished scenarios.

Consider the perception of physical elements that might be taking place around us. Each element perceived by the brain is an “output” pattern that wins a multiple hypothesis competition within a particular ISG, and these may be put into a separate class. The classes of elements might range from basic external objects (that are perceived to be the immediate sources of incoming stimuli), to actions/events (that is, relationships between, or classifications of, objects), to narratives (relationships between, or classifications of, actions/events), to generalized scenarios (relationships between, or classifications of, narratives: responses to the perception of narratives), and so on. The classes make up successive layers within a hierarchy. This is illustrated in Figure 2. We are not wedded to the number of layers shown, nor the nomenclature employed here. In this case we show four levels: objects, actions, narratives, and scenarios. The point is that the elements at each level are defined recursively in terms of the various elements from the level immediately below, and draw upon context and expectations (biases) defined from associated memories. Each element is a function of a multimodal analysis (decision-making) of the previous layer. The machinery that achieves this is identical at all successive levels—and is readily available, as we have pointed out. The recursion is very natural, robust, and repeatable, using the circuitry we have discussed above; yet it is rather hard for analysts to conceive of the consequences of this.

The vertical boxes shown in Figure 2 represent the successive pattern recognition tasks exploiting both remembered contextual information and prior expectations from past events (drawn subconsciously form memory, in an associated network fashion) as well as the assumption of the structures (elements) that are identified at the previous level. Its output is the assumption of the structures (elements) identified at the current higher level. The previous output element from the lower levels are thus the dynamic inputs to the present level. The machinery, a permissive type of Bayesian discrimination and decision-making (that we have established above, and that is readily available within the brain architecture via ISGs), is the same in each case. The elements perceived become more sophisticated and more abstract or generalized as things move up the hierarchy (to the right in Figure 2); but they all remain summaries of perceived real, external events.

For example, given our prior history in recognizing certain objects in a present context (a church service say), some visual, auditory, and olfactory stimuli, together with retrieved context and expectations, may enable us to recognize the following objects: choirboy, flame, hair, shouting, burning smell, candle. These objects together with further retrieved context and expectations allow us to recognize the following events/actions: candle burning, hair on fire, choirboy shouting. These actions together with further retrieved context and expectations allow us to recognize the following narrative: “The burning candle has set the choirboy’s hair alight, causing him to cry in pain and fear.” This narrative and further retrieved context and expectations allow us to perceive general scenarios: “an accidental emergency,” “typical misbehavior,” “an extreme event.”

There is no mystery within this process: The recursive nature of the hierarchical process and the move from objective information (the perceived real-world objects) upwards to deductions that are both more abstract and subjective mean that we find it successively more difficult to be specific about the higher domain elements.

Now consider some simple internal (mental) elements: say, mental objects such as “threat,” “arousal,” “excitement,” “amusement”; or mental feelings such as the experience of the “blueness of blue.” You cannot perceive these from any external stimuli; they are private and internal (but you might communicate them by your consequent behavior). These mental elements do not fall into a single layer within the hierarchy introduced above for physical elements. The perception of “threat” may be associated with physical objects (“flames” and “burning”), yet also with physical events (“hair setting on fire”), with the particular physical narratives, and also with the more generalized physical scenarios classifying the narratives (“an extreme event”). The mental object “threat” may trigger immediate physical responses to danger (the release of adrenalin, for example), but it must also sit within its own hierarchy of classes (layers) of mental (internal) elements.

Thus there must exist a second classification of elements that is orthogonal to the first one. Again we expect it must have multiple layers representing more and more abstract or conceptual mental elements, further away from a simple element of mental consciousness. We construct it as follows.

Consider the set *S*, containing all of the physical elements that can be perceived. Now we want to discuss how certain subsets of *S* might inspire mental elements. The natural setting for this is the *power set* of *S*, which is defined to be the set of all possible subsets of *S*. Thus any given element of the power set of *S*, usually denoted by 2^*S*^, is simply a given subset of *S*. For any finite set with *m* elements the power set is much larger, with 2^*m*^ elements; for an infinite set the power set is of a higher cardinality—since there can be no one-to-one mapping between any set and its power set.

**Figure 3.**
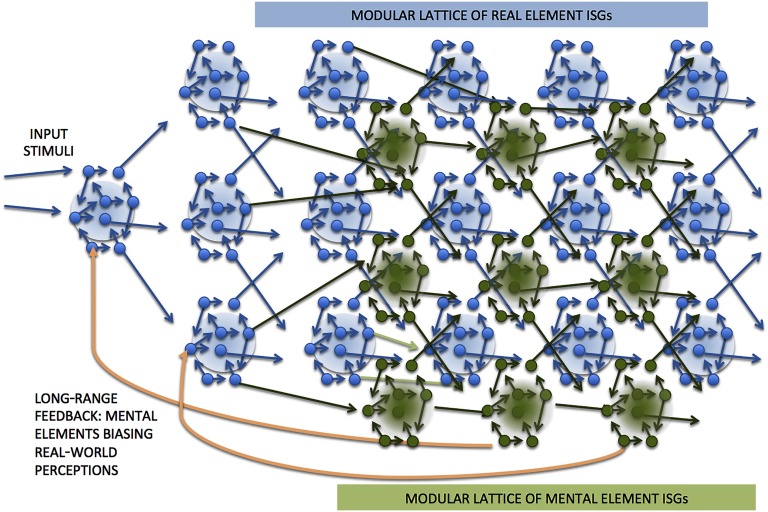
The physical elements ISG hierarchy shown in blue as a macroscopic lattice (a *network* version of the schematic shown in Figure 2); the mental elements ISG hierarchy, shown in green, is laced across the physical elements hierarchy, forming its own macro lattice, interwoven with that for the physical elements. The orange links provide long-range feedback from mental element decisions back into physical element perceptions/decisions (strictly speaking, these break the maximality of the ISGs, but we may think of these as nonlocal and acting on a longer timescale).

We assert that the set of all mental elements is isomorphic to some subset of the power set of the set of all physical elements, drawn from across any and all layers within the physical hierarchy. Each subset of physical elements constitutes a potential mental element. Alternatively we are saying that any mental element corresponds precisely to a particular subset of those physical elements (that may be drawn from across any and all layers within the physical hierarchy) that, in combination(s), invoke or inspire it, within real time. As with the physical elements hierarchy, the mental elements will also take inputs from stored contexts and expectations. These mental elements are generalized feelings and experiences. The characterization of the set of all mental elements as part of the power set of the set of all physical elements is crucial step. Note the potential mental element hierarchy is of a much larger size, or of higher cardinality, than the set of physical elements, since the latter is not isomorphic to its own power set. The situation is depicted in Figure 3, where we see mental elements represented as decision states with an array of ISGs that are laced across (conceptually orthogonal to) the array of real element ISGs.

We have argued that the power set of the set of physical elements is the appropriate structure, but others may wish to think of this a dual set, or *dual space*: that is, the set of functionals that can be defined over the set of all physical elements. At this point the difference in these concepts seems far less important than the fact that suitable candidates for the definition of the set of mental elements indeed exist. We are comfortable with either case.

Unlike the physical elements hierarchy, the mental elements hierarchy is not recursively defined. Instead we have defined mental elements in terms of the subsets (the collages) of the set of all physical elements that will (in combinations) invoke or inspire them.

Although subjective feelings and experiences (qualia) may relate obviously to some specific physical elements, they can also generalize experiences and create more abstracted connections across them. Yet they cannot be defined and communicated, or contrasted directly from brain to brain. Now, it may be argued that we are yet to give any reason to think that the mental elements in the mental hierarchy should be associated with subjective experiences. So let us reiterate and summarize the reasoning that this must indeed be so.

Why must the mental elements, defined as some subset of the power set of the set of the physical elements, be equivalent to our subjective (internal) feelings?

(a)  We have argued that feelings and experiences cannot always be placed within the physical elements hierarchy; though each may be invoked or inspired by perhaps quite a large set of physical elements, actions, events, scenarios, and so on, with some of these physical elements possibly at a high abstract level within the physical hierarchy. Therefore feelings in general must exist across the physical events hierarchy, following from a required combination or collage of physical elements that are to be perceived so as to inspire them. Similar combinations of real elements may well define similar feelings, so we may have generalized feelings (*unease*/*dread*, for example), representing classes of feelings—broad supersets, and/or sets of higher domain physical elements.
(b)  Why should sets (combinations) of physical elements define subjective feelings? Every feeling that we have ever had has been initially in response to some original combination of external physical events: these (first-time experience) physical elements together defined the feeling, within the moment. Indeed if we reperceive, or remember, the right set of corresponding physical elements (a specific event, a situation, an image, a fact, a narrative, and so on), in the right combinations or collages, then we can reexperience the desired mental feeling. We can make ourselves feel sick with disgust, or shocked, or filled with awe. But we do not do so in the abstract (we do not will ourselves to feel something and flick a mental switch): We must mentally reconjure some combination of physical elements. With practice we might become very proficient at this and also possibly hardened to certain experiences (reducing the initial physical reactions to them). Therefore our feelings are indeed invoked or inspired by certain collages of physical elements (even if they are not all actually present, but imagined). They are really just a classification of combinations of physical elements (some of which might be abstract and complex—relations between relations between relations).
(c)  Why must all feelings be inspired in this way, and be defined in terms of a combination of physical elements? Put more simply: Can we have a feeling that is not well defined in this way? Can we have a feeling that requires anything else, additional? Sometimes feelings can be combinations of other feelings; but that does not count as an exception. Suppose now that there is a particular feeling that is contingent on something other than a set of physical elements (or mental feelings that are already defined in terms of physical elements). How did that experience come about inside our minds for the first time; what precipitated it? There must have been a set of circumstances and physical stimuli, from our own senses or the workings of our own body (as an organism), that communicated and instigated some information, as patterns of neuronal behavior, into our brain. But this information *is* physical and it *defines* physical elements. If there were anything else it would have to produce some spontaneous patterns of firing behavior across some or many ISGs. As nonphysical this pattern formation might be internally created, such as by in instability-driven pattern formation, like Turing patterns, following spontaneous symmetry breaking within certain modes, or as a last resort, by noise (chatter between neurons). We cannot rule this out; but either of these would be uncontrolled aberrant firing patterns in time and across the cortex, created by random perturbations and instabilities; or noise within the system. Yet these can not give rise to *feelings* as they plainly cannot be reconjured at will. They are akin to functional breakdowns (aberrations) rather than facets of the organism’s regular experiences. So let us restrict our definition of feelings to those subjective experiences that can be relived at will. Then we must discount internal instability-driven pattern generation and noise as causal elements of feelings.

Of course any individual may or may not have perceived any particular mental elements (as yet). They may or may not have perceived the requisite combination (subset) of the physical elements. So each organism will have its own version of the mental element hierarchy depending on the sophistication of its real experiences, as held within its current version of the physical element hierarchy. We must understand that both sets, that of perceived physical elements, and that of the induced mental elements, are constantly growing with the experience and the familiarity of experiences or the organism.

There is no *extra ingredient* here: The processing is applied recursively and is updated at all levels within the real element hierarchy. Both hierarchies can be updated simultaneously. However, the dual hierarchical model certainly helps us to understand how elements become more remote from the simple real elements (low-domain real objects and events) and the simple mental elements (induced by just a few low-domain real objects and/or events), and hence they become more subjective and abstracted, while also being as “real” to the brain as are much lower domain objects and events.

There are always more layers that might be instantiated in the hierarchies, possibly a countable infinity of layers due to the recursive definition in both hierarchies. As the layers of elements become more abstract and more subjective (and experiential), there may be many layers that are harder and harder to discern from one another. It will almost certainly be the case that there are higher layers in the mental hierarchy representing super-experiences, which may emerge from very distinct experiences and specific exposures of the organism to different types of situations (the mental elements hierarchy might be itself constantly developing and is not assumed to be hardwired with a fixed number of levels). Some of these super-experiences may be developed by individuals with specific exposure and practice that is unusual for others (such as experts in perfume, taste, and music). It may be that certain claimed religious experiences gained via meditation or other activities are simply developing and accessing these higher and higher layers of experience within the hierarchy. Thus all similar organisms may have the potential to develop more and more super-experience (higher domain) layers within our proposed mental hierarchy; it is very likely that only the lower levels will be most common (up to and including common experiences, common qualia). Yet certain individuals, through their own effort or exposure, may develop such super-experience layers of processing that are accessible for them as a consequence of certain types of stimuli and internal (biased) retrieval.

The high-level mental elements such as feelings or experiences may feed back into the bias or control of the retrieval of contextual information and prior expectations that are drawn into the pattern recognition and multimodal decision-making earlier down within both hierarchies (as depicted in the orange feedback connections in Figure 3). Thus the whole has a feedback loop: If we experience feelings of ecstasy or rage, then that will constrain and bias the kind of information we retrieve in clarifying the present context. Thus recognized elements at low levels will be updated (dynamically) partly in light of the perceived higher domain elements.

Notice that if the set of real elements is finite, bounded once and for all with say at most *n* of them, then so would the set of mental elements: There would be at most 2^*n*^ of them. Then a robot or zombie could learn all of them by rote. The extent of the mental hierarchy will be dependent on the individual’s experience. It may be that some brains have highly discriminating feelings within some fields of regular exposure or expertise, and that these might be refined through effort. The mental hierarchy evolves and cannot be embedded or learned a priori.

## FURTHER CONSIDERATIONS

It is straightforward to imagine how the “physical” element ISGs (those making modal decisions between physical elements) might be wired together so as to provide for recursive and hierarchical decision-making for the higher and higher domain (more abstract) physical elements. Then the mental elements could be modes exhibited by “dual” ISGs that are arranged across these, taking inputs from various physical ISG modes. However, we also want the mental elements—dynamical modes exhibited by the dual ISGs—to provide some feedback (biases) into the near-term future workings of the physical elements hierarchy. For example, once a organism is in *panic* mode, then this may color even basic perceptions of incoming physical objects and events. And, of course, love is blind. Such feedback, necessary for rapid response (thinking fast in Kahneman, 2011) and top-down biasing or priming, must mean that there are some longish (thus slow) connections back from the dual ISGs into the physical element ISGs. Of course such feedback formally violates the idea that ISGs are maximal (and thus dynamically isolated from any downstream processing); the neurons within the dual ISGs are both upstream and downstream of some of the physical element ISGs. Recall that the main point of ISGs is that they should develop dynamical modes (make decisions) on relatively fast timescales; that can still happen via short path circuits on a short, local timescale so long as any feedback involves a longish path (relative to the circuits within ISGs) and thus operates on a timescale that is longer. The idea that the tight, local ISGs are maximal will thus need to be relaxed a little so as to allow such obvious feedback, with the mental modes and feelings biasing even basic physical perceptions via longer range path on (an order of magnitude) longer timescales. This is shown in Figure 3.

Some connections within and across ISGs will be much more useful than others. These well-worn paths can be strengthened through use, while the lesser employed connections may diminish. There is thus some natural tuning, and it is biased around what is commonly experienced. It is also possible that modes from many distinct physical element ISGs that almost always occur together (out of many millions of ISGs) may be laced together as co-inputs to an appropriate dual ISG. Thus usage (and repetitive learning) and plasticity may go a very long way to shaping the initially large population behavior of ISGs into efficient bundles. The point is that it is a coincident and collective response across both small and large subpopulations of ISGs that is essential to the development of the dual architecture and global processing.

The heterogeneity of the neuron-to-neuron connections, thus the ISGs, appears to be essential. If all ISGs were the same (with the same structure, same transmission lags, and so on) and these were wired uniformly on a *grid*, then dynamical experiments show that the dimension of dynamical behaviors obtained collapses down very radically. The brain has no need to achieve any such uniformity and indeed appears to make a virtue of some heterogeneity and randomness, directly increasing processing capacity.

What is expensive here for the organism? The dual ISGs, discriminating between mental elements (feelings), might grow and grow compared with the physical elements. It is obvious to speculate that a physical hierarchy with relatively simply primitive feelings across it might be instantiated rather early in the developing brain, while the more and more nuanced mental elements could come much later and be biased by the organism’s experiences and requirements. A learning brain may even eventually reuse or redeploy circuits to increase the processing capacity for mental elements.

It is interesting to think about the neurons themselves making new connections over time within the proposed architecture. The phenomenon of neuroplasticity allows the brain not only to compensate for failures, but also to develop higher level perceptive abilities and feelings. From the point of view of the (loosely connected) ISGs, it is clear that new connections should follow a *Goldilocks principle*. Further connections that are very short range, and hence have very short time lags, will only result in local cycles that are too fast and not sustainable because of the refractory nature of firings; connections over long ranges with long time lags result in long transients before the ISG could reach a responsive equilibrium (longish rate limiting connections) and thus induce inefficiencies. ISGs could best develop by having medium-scale connections increasing their size (*n*) incrementally. Of course, the mental elements are determined by ISGs that may have long-range inputs from various distributed (physical element) ISGs. We would expect that such development may take a long time (years).

## FEELINGS AS LATENT VARIABLES

Why do we need a mental elements hierarchy? Why can we not all appreciate zombies, relying merely upon the physical elements hierarchy and learning to make it as complete as possible? (In philosophy, zombies are imaginary creatures that are exactly like humans in all physical respects but without any conscious experiences, such as feelings.) We respond by considering analogous constructs in efficient problem solving.

The mental elements are equivalent to unobservable *latent variables* deployed within multihypothesis decision-making, multimode dynamics, and control problems. Without such latent modes, employed in order to switch from one behavioral mode to another, say from flight to fight, an organism would need a huge amount of physical evidence, and would need to consider huge numbers of hypotheses (“What can happen next?”).

The *latency* concept is fundamental within hidden Markov models, for example. There a system behaves according to its *state*, which at any time is in one of a discrete number of alternatives. Depending on its state the system will behave in a certain way, governed by internal “latent” variables, in producing output based on present inputs. When the system transitions from one state to another, its mode of operation changes and it may be observable, under uncertainty, in terms of its input-output behavior. There is much research in reverse engineering such hidden Markov models in various applications (the EM algorithm, mentioned earlier, can be used to infer and distinguish latent state labels). The existence and switching of the latent (hidden) states would help achieve whole organism behavioral changes far more quickly than some bottom-up reasoning. It also biases or focuses the system’s response to certain cases of inputs, and hence produces certain classes of outputs.

So in order to be effective and efficient, even zombies or computerized robot minds would best include a rather wide range of latent states, operating over both small and large domains. Indeed, dual systems are prevalent throughout both control (for example, of Hamiltonian systems and bang-bang control theory) and optimisation theory (such as in linear programming and so on). Often it is far more efficient to resolve issues within the dual system (in our case the dual hierarchy) to drive and focus the physical one. We contend that the dual hierarchy system evolves to serve this purpose and is embedded within the architecture as opposed to being instantiated in processing work.

Feelings might play the same role as latent variables, by reducing the current focus (the feasible set) for decision-making, biasing the perception of external objects and events, and enabling faster responses by reducing the whole set of possible situations and events that may occur. This would also encourage social interactions (when organisms are in similar states of behavior for the time being).

Our suggestion is that the process of evolution would consign advantages to organisms capable of resolving problems within such a dual system approach, making up for incomplete information and resolving behavioral response rapidly and top down (in the mental) at the same time as bottom up (in the physical), in perceiving what is physically happening. The identification of fast-thinking heuristics (“What you see is all there is;” Kahneman, 2011) supports this idea. This last is really a *nonconstructive argument for the existence of a dual hierarchy* (contrasting with the constructive basis given in the previous sections).

## SUMMARY: IMPLICATIONS OF THE DUAL HIERARCHY MODEL

An interesting (and perhaps crucial) difficulty is one of counting (of making lists). If we limit a mental element *Y* to be equivalent to a finite set of justifying or associated elements, then we fail: A zombie or a robot could easily learn all of those elements in *Y* by rote (given time). There would be nothing there in *Y* that “Mary didn’t know” (Jackson, 1986; Jackson introduced Mary in a thought experiment to show that physical information about the world, and of colors in particular, cannot convey the human experience of actually perceiving things). Yet if an *ideal Y* includes an uncountable number of nuanced elements, with no a priori bound, then even a lifetime could never be enough to list or assemble them all (and there must always be some nonphysical nuances within *Y* that “Mary didn’t know”). Those mental states corresponding to a countable infinity of physical elements lie beyond the learning abilities of robots or zombies (or Mary). Such states must be experienced and perceived more and more subtly through an organism’s own recursive processes and the mapping from the physical hierarchy onto the mental hierarchy. Of course it is also possible that the elements, and thus levels within the hierarchies, might become unlearned—perhaps as a result of cognitive decline or else a lack of continual usage.

The boundedness of the mind is an important factor, as human capacity is certainly bounded in terms of neurons. The idea of having firing patterns (modes for ISGs) characterized *both* in time and across the ISG’s neurons makes those bounds very, very, large though. But the effective capacity to discriminate between experiences remains practically finite. Boundedness for humans with allocation and reallocation of resources (learning and unlearning) is less serious than that for the zombies (the equivalent of humans but without feelings): Humans develop expertise and thus some refined qualia within whatever fields most useful and most common. Zombies will be bottom-up deciders and will be slow thinkers (deprived of fast thinking), where everything they have ever experienced is always a possibility within the next instance. Thus feeling/internal elements are the things that confer an evolutionary advantage to a human brain (over such zombies).

Though the dual hierarchy model is infinite in concept, in practice every organism is on a expanding (or contracting) journey. At any moment there are always experiences that have not been felt and are as yet unknown to each organism. The difference between a human brain and a zombie is that the brain has the potential to include any mental elements, as appropriate, over time, whereas the zombie mind is bounded a priori and constrained to the real element hierarchy. For the avoidance of doubt we do not propose that a brain could exploit the recursive machinery at its own convenience and apply it to generate real elements on the fly as desired; recall that the recursion is instituted and hardwired with connections between ISGs.

A human brain is continuously learning and experiencing, and so it has the potential at least in time to become more and more conscious, and to reach higher and higher domains. Consciousness is thus a relative concept and is best spoken of as a journey within the dual hierarchy rather than as an achievable destination.

The “explanatory gap,” often discussed within philosophy, refers to the division between information about the real world (and about how a human brain functions), and the subjective experiences of internal phenomena, what it feel like to experience something within the real world (the roughness of a surface or smell of an animal). Such phenomenal aspects are referred to as “qualia.” We have argued that both the physical elements and the mental elements (feelings, qualia) are induced by the same mechanisms. They are both supported by, and are a consequence of, the same neural architecture of loosely coupled ISGs.

We emphasize that for some feelings there may be no finite listing and thus no full learning of everything that is associated with that subjective experience. Each individual is working though a possibly longer and longer list of learning about what such an experience involves (what it is like to experience “blue”); but it will never be complete, even in a lifetime. Thus the “explanatory gap” might better refer to what is achievable by an evolving individual brain, going through any (finite) learning and experience, compared with the infinite *whole nine yards*, which none of us will ever experience. There may thus always be nuanced qualia that a given individual is yet to experience.

It is therefore not for us, in proposing that this candidate model structure (both dynamics and architecture) is capable of reflecting all facets of mental subjective feelings (even infinitely many), to show that the mental elements correspond to subjective feelings; but it is for those challengers of the model to bring forward any one single facet of any subjective experience which could not be represented within the dual hierarchy, instantiated in the neural architecture and dynamics, and thus experienced at some point by organisms that are learning and growing thus.

The dual hierarchy model is but one of a number of *straw man* models that can be founded on our knowledge of the dynamical behavior of many loosely connected tight bundles of neurons (ISGs). In addressing the hard problem we have discussed how feasible the instantiation of mental elements would be within a human brain evolving to become an efficient and effective decision-maker in the presence of rapid, incomplete, incoming stimuli. Mathematics is far from ready to concede ground to the strong emergence concept; and indeed the term “emergence” applied to conscious phenomena is somewhat of a red herring. There is much that we can understand now and in the future by *modeling out* from that which is already known.

## ACKNOWLEDGMENTS

I am grateful for support from a number of EPSRC research grants: EP/G065802/1, *The Digital Economy HORIZON Hub*; EP/I017321/1, *MOLTEN: Mathematics of Large Technological Evolving Networks*; EP/F033036/1, *Cognitive Systems Science (Bridging the Gaps)*; EP/I016856/1, *NeuroCloud*; and EP/H024883/1, *Towards an Integrated Neural Field Computational Model of the Brain*. I am also pleased to acknowledge the advice and encouragement of my colleagues on those grants, especially Doug Saddy, Des Higham, and Clive Bowman. Thanks to David Chalmers and Karl Friston for very constructive advice. Finally I would like to thank the anonymous referee who helped with the structure and presentation of this paper.

## AUTHOR CONTRIBUTIONS

Peter Grindrod: Conceptualization; Formal analysis; Visualization; Writing – original draft. Writing – review & editing.

## TECHNICAL TERMS

Strongly connected delay networks: Networks or subnetworks where there is a directed path between any ordered pair of vertices, and each edge incurs some time delay in the passage of information (spikes).
Dual hierarchy: An architecture with one hierarchy orthogonal to the other.
Strongly connected delay networks: Networks or subnetworks where there is a directed path between any ordered pair of vertices, and each edge incurs some time delay in the passage of information (spikes).
Bayesian nonbinary processing: This uses Bayesian updating to adjust the probabilistic support for a range of mutually exclusive hypotheses. Such hypotheses compete until a decision is required where the winning outcome is the hypothesis with the highest current probability.
Latent variables: Hidden variables, not observable as state variables, which may need to be inferred and which can make decision and optimization problems easier by reducing the relevant information required.
